# Preserflo™ microshunt for the treatment of refractory childhood glaucoma: A case series study

**DOI:** 10.1016/j.ajoc.2025.102384

**Published:** 2025-07-08

**Authors:** Yuri Tanaka, Shiho Kasahara, Ryo Tomita, Norie Nonobe, Kazuhide Kawase, Kenya Yuki

**Affiliations:** a Department of Ophthalmology, Nagoya University Graduate School of Medicine, 65, Tsurumai, Syowa Ward, Nagoya, Aichi, 466-8560, Japan; b Ito Eye Clinic, 25-1, Gonaka, Azaicho, Nishiazai, Ichinomiya, Aichi, 491-0113, Japan; c Yasuma Eye Clinic, 4-10-50, Osu, Naka Ward, Nagoya, Aichi, 460-0011, Japan

**Keywords:** Anti-glaucoma medications, Childhood glaucoma, Intraocular pressure, Preserflo™ microshunt, Trabeculotomy

## Abstract

**Purpose:**

We report the outcomes of five patients with pediatric glaucoma, who were treated with Preserflo™ microshunt surgery.

**Methods:**

This prospective study included five consecutive patients who underwent Preserflo™ microshunt surgery at the Nagoya University Hospital. The main outcome measures evaluated in this study were mean intraocular pressure (IOP), number of glaucoma medications, and best corrected visual acuity (BCVA). Paired t-tests with Bonferroni correction were used to compare the baseline and follow-up periods, where p < 0.05 was considered statistically significant.

**Results:**

Average age at the time of surgery was 10 ± 4 years. Three patients had primary congenital glaucoma, one had Sturge-Weber syndrome, and one had juvenile open-angle glaucoma. Preoperative average IOP was 37.6 ± 4.7 mmHg. After microshunt surgery, IOP significantly decreased to 6.3 ± 1.2 mmHg in 1 day, 6.6 ± 1.7 mmHg in 1 week, 9.6 ± 6.1 mmHg in 1 month, 12.0 ± 7.4 mmHg in 3 months, 10.0 ± 5.2 mmHg in 6 months, 14.2 ± 8.7 mmHg in 9 months, and 10.5 ± 6.5 mmHg in 1 year (p < 0.01 for each paired *t*-test). One year postoperatively, we placed a Baerveldt glaucoma implant in case 1. No significant change was observed between the pre- and post-operative periods in BCVA. No postoperative complications, including choroidal detachment, shallow or flat anterior chamber, hypotony maculopathy, or conjunctival leak, were observed in this case series.

**Conclusion:**

Microshunt surgery may be a useful option for treating glaucoma in pediatric patients, in whom trabeculotomy failed.

## Highlights

• Preserflo™ Microshunt reduced intraocular pressure in refractory childhood glaucoma.
• Four of the five patients were free from additional glaucoma surgery at 1year following Preserflo™ microshunt surgery.
• No severe complications were observed during and after Preserflo™ microshunt surgery.

## 1 Introduction

Primary congenital glaucoma is a rare disease with an incidence of 4.8 per 100000 live-born in Denmark, and 5.4 per 100000 live-born in the United Kingdom.^1^^,^^2^ In primary congenital glaucoma, the main mechanism is considered to be increased aqueous humor outflow resistance due to trabecular meshwork dysgenesis and high iris insertion, resulting in intraocular pressure (IOP) elevation.^3^^,^^4^ Hence, incising the trabecular meshwork can be effective for primary congenital glaucoma.^5^ Surgery to incise the trabecular meshwork such as ab externo trabeculotomy, ab interno trabeculotomy, goniotomy, or gonioscopy-associated trabeculotomy, has been shown to be effective for primary congenital glaucoma.^6^ Ikeda et al. reported that the IOP of 86 % patients with primary congenital glaucoma normalized after the first ab externo trabeculotomy.^5^ Ikeda et al. also reported that only four (3.5 %) of 112 eyes with primary developmental glaucoma, who underwent one to three trabeculotomies, required trabeculectomy or cyclocryotherapy.^5^ After several trabeculotomies for childhood glaucoma, further glaucoma surgical options such as trabeculectomy, glaucoma drainage implant, or cyclophotocoagulation are available.

A microshunt (Preserflo™ microshunt, Santen Pharmaceutical Co. Ltd., Osaka, Japan) surgery was approved for use in Japan in 2022. Microshunt is composed of styrene-block-isobutylene-block-styrene—which is a flexible and highly biocompatible synthetic polymer—and has a length of 8.5 mm, outer diameter of 350 μm, and inner lumen size of 75 μm. In a randomized controlled trial, microshunt significantly reduced IOP, although the postoperative IOP was higher than trabeculectomy in adult patients with primary open-angle glaucoma (POAG).^7^ Also, long-term safety and efficacy have been reported in Japanese patients with POAG.^8^ Pillunat et al. reported that the rate of interventions after surgery was significantly lower in the microshunt group compared with that in the trabeculectomy group in adult glaucoma patients.^9^ In this study, during the first postoperative 6 months, intervention was required in 5 patients out of 26 patients (19 %) in the microshunt group, and 16 patients out of 26 patients (62 %) in the trabeculectomy group, which was a statistically significant difference (p = 0.004, Fisher's exact test). Secondary interventions such as removal of the releasable suture, conjunctival suture for conjunctival leak, bleb needling, and anterior chamber reformation for flat anterior chamber cannot be completely avoided after trabeculectomy. In children, it is difficult to perform these procedures under local anesthesia, and general anesthesia is required each time a secondary intervention is required. Microshunts, therefore, may be suitable for the treatment of childhood glaucoma. Herein, we report the results of five cases of refractory childhood glaucoma treated with microshunt surgery.

## 2 Methods

This prospective study was conducted in accordance with the tenets of the Declaration of Helsinki, and was approved by the Ethics Committee of Nagoya University Graduate School of Medicine (approval number #2023–023929934) and was preregistered in the University Hospital Medical Information Network (UMIN) Clinical Trials Registry (UMIN000050608; https://www.umin.ac.jp/ctr/index.htm). Written informed consent was obtained from all the study participants’ parents prior to surgery.

### 2.1 Microshunt procedure

One surgeon (KY) performed all surgeries. After general anesthesia and routine disinfection, a superior nasal or superior temporal fornix-based conjunctival flap was created, and 2 % lidocaine was placed under the Tenon space. The surgeon usually places the microshunt superonasal for the right eye, and superotemporal for the left eye because the surgeon is right-handed. The conjunctiva and Tenon's capsules were carefully dissected to the sclera. After hemostasis, a sponge soaked in mitomycin C (0.4 mg/mL, Kyowa Kirin Co. Ltd., Tokyo, Japan) was applied to the quadrant under the Tenon's layer for 5 min with Ben Sheets (Kawamoto-Sangyo, Osaka, Japan).^10^ The ocular surface and conjunctiva were irrigated using 100 ml of balanced salt solution. A scleral tunnel, marked 3 mm from the limbus, was created into the anterior chamber using a double-step knife. Subsequently, a microshunt was inserted through the tunnel into the anterior chamber. After ensuring that it was located above the iris, balanced salt solution was injected into the microshunt to ensure that fluid flowed through the distal end of the device. We injected balanced salt solution from the posterior end of the microshunt using the perfusion needle supplied with the kit. The posterior end of the microshunt was sutured using 10-0 nylon (Ethilon™; ETHICON, Ohio, United States of America (USA)) and lightly pressed against the sclera. The 10-0 nylon was placed approximately 1.5 mm from the posterior end of the microshunt, in the middle of the distal portion. Tenon's layer and conjunctiva were carefully closed together using 8-0 vicryl (ETHICON, Ohio, USA) to ensure watertight closure of the conjunctiva with two wing mattress sutures. Postoperatively, topical antibiotics (1.5 % levofloxacin hydrate) and steroids (0.1 % betamethasone sodium phosphate) were administered four times daily for 4 weeks.

Information collected for each patient before surgery included age, sex, type of glaucoma, anti-glaucoma medications, type of glaucoma surgery that patients underwent before microshunt surgery, history of intraocular surgery, and preoperative IOP. All patients were hospitalized postoperatively and observed by the ophthalmologist who performed the surgeries. All patients were discharged the day after microshunt surgery. Postoperative slit-lamp microscopic examination, IOP measurements, best corrected visual acuity (BCVA, LogMAR) measurements, and funduscopic examinations were performed at 1, 2, 3, 4, 5, 6, 9, and 12 months. Corneal endothelial density could not be measured in all the cases. IOP was measured using iCare IC100, or iCare (M.E.Technica INC, Tokyo, Japan).

### 2.2 Inclusion and exclusion criteria

Five consecutive patients with refractory childhood glaucoma following previous glaucoma surgery who underwent microshunt insertion at the Nagoya University Hospital between December 2023 and March 2024 were included. No exclusion criteria were applied.

### 2.3 Main outcomes

The main outcome measures evaluated in this study were the mean IOP, number of pre-and post-operative glaucoma medications, and BCVA. Paired t-tests and Bonferroni corrections were used to compare baseline and follow-up periods. Probability values < 0.05 were considered significant. All data were analyzed using Statistical Package for the Social Sciences (SPSS, v29, IBM Corp., Armonk, NY, USA).

## 3 Results

The demographic characteristics of the patients are shown in Table 1. Four of the five patients were male, and one was female. The mean age at the time of surgery was 10 ± 4 years. Three patients had primary congenital glaucoma, one had Sturge-Weber syndrome, and one had juvenile open-angle glaucoma. All patients had clear corneas, and were phakic. In Cases 1 to 4, the microshunt was placed supra-nasally in the right eye, and in Case 5, the microshunt was placed supra-temporally in the left eye. Preoperative average IOP was 37.6 ± 4.7 mmHg. After microshunt surgery, IOP significantly decreased to 6.3 ± 1.2 mmHg in 1 day, 6.6 ± 1.7 mmHg in 1 week, 9.6 ± 6.1 mmHg in 1 month, 12.0 ± 7.4 mmHg in 3 months, 10.0 ± 5.2 mmHg in 6 months, 14.2 ± 8.7 mmHg in 9 months, and 10.5 ± 6.5 mmHg in 1 year (p < 0.01 for each paired *t*-test, IOP data of Case 1 was omitted at the 1 year time point because a glaucoma drainage device (GDD) was implanted at 12 months after microshunt surgery). IOP data was included after cases 1 and 2 started glaucoma medications. Case 1 was initiated on latanoprost/carteolol combination drug and ripasudil 9 months after microshunt surgery, because the IOP increased to 26 mmHg. The IOP in Case 1 increased to 30 mmHg with latanoprost/carteolol combination drug and ripasudil 11 months after microshunt surgery. Thus a Baerveldt glaucoma implant was placed in this patient 12 months after microshunt surgery. Case 2 was initiated on latanoprost and timolol/dorzolamide combination drug 12 months after microshunt surgery, because the IOP increased to 28 mmHg.

**Table 1:** Demographic and patient characteristics among 5 children with refractive childhood glaucoma who underwent microshunt surgery.

Case	Age (y)	Sex (M/F)	R/L	Type of glaucoma	Bi/Uni	Family history of glaucoma	Previous history of trabeculotomy, times (type)	History of MPCPC (times)	Pre/Post-operative IOP (mmHg)	Pre/Post-operative number of topical glaucoma medications	Pre/Post-operative BCVA (LogMAR)
1	4	M	R	PCG	Bi	+ Sibling	2 (ab externo)	1	38/30	4/4	1.3/1.5
2	6	M	R	SWS	Uni	–	2 (ab externo)	0	39/20	4/3	1.7/1.5
3	12	M	R	PCG	Bi	–	3 (ab externo)	2	38/9	5/0	0.7/0.5
4	12	F	R	PCG	Bi	+ Mother	3 (ab externo)	2	30/7	5/0	0.7/1.0
5	14	M	L	JOAG	Bi	–	1 (s-lot ab interno)	0	43/6	5/0	1.4/1.4

Abbreviations: BCVA, best corrected visual acuity; Bi, bilateral; F, female; IOP, intraocular pressure; JOAG, juvenile open-angle glaucoma; L, left; M, male; MPCPC, micro-pulse cyclo-photocoagulation; PCG, primary congenital glaucoma; R, right; SWS, Sturge-Weber syndrome; s-lot, 360° suture trabeculotomy; Uni, unilateral.Post-operative values were obtained at 12 months after microshunt surgery, except for Case 1, in which they were recorded at 12 months just before Baerveldt glaucoma implant surgery.

Preoperative average BCVA was 1.2 ± 0.4, and 1.3 ± 0.1, 1.1 ± 0.3, 1.2 ± 0.3, and 1.2 ± 0.3 at 3, 6, 9, and 12 months after microshunt surgery, respectively. No significant change was observed between the pre- and post-operative periods in BCVA (p = 0.99, paired *t*-test).

No intra- or post-operative complications were observed, including shallow or flat anterior chamber, choroidal detachment, hypotony maculopathy, or conjunctival leak. Of note, post-operatively, cases 1 and 2 were uncooperative with the ophthalmic examination, and we could not examine them in detail.

## 4 Discussion

In this study, four of five patients were able to maintain IOP normalization for at least 1 year following microshunt surgery for refractory childhood glaucoma. Three of the five patients achieved an IOP less than 15 mmHg without glaucoma medication. Only one patient needed additional glaucoma surgery 1 year after microshunt implantation.

A prior report on microshunt surgery for childhood glaucoma revealed that in 12 patients with a history of previous glaucoma surgery, including angle surgery with subsequent GDD (n = 5), GDD alone (n = 3), and ab interno angle surgery alone (n = 4), after microshunt surgery 75 % (9/12) of these patients achieved success with 7/9 taking no medications, 2/9 requiring 2 medications, and only 3/12 requiring additional surgery at 1 year.^11^ Furthermore, they also reported a significant decrease in IOP from 22.7 ± 4.8 mmHg preoperatively to 11.9 ± 3.8 mmHg after 1 year of surgery. In our study, four of five patients (4/5, 80 %) were able to maintain IOP normalization for more than 1 year. Microshunt surgery is a surgical option that needs further evaluation based on our promising pilot study data.

The Ahmed glaucoma valve or Baerveldt glaucoma implant could be an option for childhood glaucoma. Both techniques have been reported to be effective for childhood glaucoma.^12^ Insertion of the Ahmed glaucoma valve and Baerveldt glaucoma implant into the anterior chamber have been associated with a significant decrease in corneal endothelial cell density 20 months after surgery^13^ and 5 years after surgery.^14^ However, microshunt has been reported to cause no significant decrease in corneal endothelial cells density in adult patients 2 years after surgery,^15^ or is comparable to trabeculectomy 2 years after surgery.^16^ Future reports are needed to determine whether GDD or microshunt is less harmful for childhood glaucoma with respect to corneal endothelial cells.

This study has a few limitations. Since this case series has only five cases, we cannot apply this result to pediatric glaucoma as a whole. In addition, this study did not compare microshunt surgery with glaucoma drainage implants or trabeculectomies. Trabeculectomy has been reported to be superior to microshunt surgery in lowering IOP in adults with glaucoma.^16^ The results of this study were short-term and obtained at 1 year postoperatively. The effect of microshunt surgery on corneal endothelial cells in childhood glaucoma remains unclear. Future studies in a large sample with longer follow-up period comparing GDD and trabeculectomy to microshunt in pediatric glaucoma are required to validate the findings of this study.

## CRediT authorship contribution statement

**Yuri Tanaka:** Resources, Methodology, Investigation. **Shiho Kasahara:** Writing – original draft, Visualization, Methodology, Investigation, Conceptualization. **Ryo Tomita:** Supervision, Methodology, Data curation, Conceptualization. **Norie Nonobe:** Resources, Methodology, Conceptualization. **Kazuhide Kawase:** Writing – review & editing, Supervision. **Kenya Yuki:** Writing – review & editing, Writing – original draft, Visualization, Validation, Supervision, Software, Resources, Project administration, Methodology, Investigation, Funding acquisition, Formal analysis, Data curation, Conceptualization.

## Patient consent

Consent to publish this case report has been obtained from the patients in writing. This report does not contain any personal identifying information.

## Declaration of competing interest

The authors declare that they have no known competing financial interests or personal relationships that could have appeared to influence the work reported in this paper.
